# Incidence and Outcomes of Unplanned Intensive Interventions After Common Inpatient Surgeries: A Population-Based Cohort Study

**DOI:** 10.1097/AS9.0000000000000692

**Published:** 2026-07-03

**Authors:** Samir K. Shah, Lingwei Xiang, Rachel R Adler, Martin Viola, Zara Cooper, Clancy J. Clark, John Hsu, Emily Finlayson, Dae Hyun Kim, Kueiyu Joshua Lin, Joel S. Weissman

**Affiliations:** From the * Division of Vascular Surgery, University of Florida, Gainesville, FL; † Center for Surgery and Public Health, Brigham and Women’s Hospital, Boston, MA; ‡ Center for Surgery and Public Health, Brigham and Women’s Hospital, Boston, MA; § Division of Surgical Oncology, Virginia Mason, Seattle, WA; ¶ Department of Health Care Policy, Harvard Medical School, Mongan Institute, Massachusetts General Hospital, Boston, MA; ‖ Department of Surgery, Phillip R. Lee Institute for Health Policy Studies, University of California, San Francisco, CA; ** Marcus Institute for Aging Research, Hebrew SeniorLife, Boston, MA; †† Division of Pharmacoepidemiology and Pharmacoeconomics, Department of Medicine, Brigham and Women’s Hospital, Boston, MA.

**Keywords:** intensive interventions, Medicare claims, surgical outcomes

## Abstract

**Objective:**

To determine the incidence, predictors, and outcomes of unplanned intensive interventions after common inpatient surgeries among older adults.

**Background:**

Intensive interventions in older adults may profoundly affect prognosis and recovery after surgery, yet their frequency and association with outcomes such as mortality are unknown.

**Methods:**

We conducted a retrospective cohort study using Medicare fee-for-service claims (2016–2020), including patients aged ≥66 years who underwent 1 of the 10 most common inpatient surgeries. Unplanned intensive interventions—cardiopulmonary resuscitation, prolonged mechanical ventilation, tracheostomy, new dialysis, extracorporeal membrane oxygenation, or feeding tube placement—were identified during the index hospitalization. Multivariable generalized estimating equation models identified predictors, and propensity score-weighted models assessed 1-year outcomes.

**Results:**

Among 1,095,445 surgeries, intensive interventions occurred in 20,669 (1.9%) patients, most commonly cardiopulmonary resuscitation (67.6%) and prolonged ventilation (22.3%). Frailty was the strongest predictor [adjusted odds ratio (aOR) 1.67, 95% confidence interval (CI) 1.60–1.73], followed by urgent/emergent surgery (aOR 1.18, 95% CI 1.13–1.23), older age, male sex, and higher comorbidity burden. Patients experiencing intensive interventions had markedly worse outcomes: inpatient mortality 24.6% versus 0.5% (aOR 51.4, 95% CI 47.1–56.1), 1-year mortality 44.2% versus 5.2% (aOR 11.6, 95% CI 10.8–12.4), and 90-day nursing home admission 8.9% versus 1.8% (aOR 5.0, 95% CI 4.2–5.9).

**Conclusions:**

Although uncommon, unplanned intensive interventions after common surgeries are associated with dramatically higher mortality and institutionalization. Frailty, comorbidity, older age, male sex, and urgent/emergent surgery identify patients at greatest risk. These findings support incorporating intensive intervention risk into preoperative discussions and postintervention care planning to promote goal-concordant decisions.

## INTRODUCTION

Surgery is commonplace among older adults: for Medicare beneficiaries ≥65 years, the likelihood of at least 1 major operation in a 5-year period is approximately 1 in 7, resulting in roughly 4 million operations annually.^1,2^ When older adults decide whether to proceed with surgery, knowledge of the likelihood and severity of adverse postoperative outcomes consistently ranks among their top information needs.^3^ Expectations about prognosis do not simply inform patients; they shape choices. For example, hemodialysis patients who over- or underestimate survival prospects make different dialysis modality and advance-care decisions, and patients with metastatic lung cancer who overestimate survival are more likely to pursue aggressive treatment.^4,5^ Yet surgeons and shared decision-making tools often distill risk into overly broad endpoints (eg, “any complication”), masking differences that matters to patients. Not all complications are equal and, among the hundreds of possible postoperative complications, those that require potentially life-sustaining “intensive interventions” (eg, new long-term dialysis or cardiopulmonary resuscitation) may disproportionately influence surgical choices and broader goals of care for an array of reasons, including impact on quality of life.^6,7^ For example, patients and families may ask about the implications on survival and return to home after the occurrence of postoperative cardiopulmonary resuscitation (CPR) to guide care decisions. Understanding the incidence and consequences of intensive interventions is important for truly informed consent and goal-concordant perioperative decision-making.

Although intensive interventions have been operationalized by others for studies of end-of-life care, this has not been done as intermediate outcomes for common inpatient surgical procedures spanning cardiac, orthopedic, and general surgical specialties.^8,9^ Although these 6 interventions vary widely in invasiveness and acuity, they share a common attribute—each is unplanned, potentially life-sustaining, and burdensome—and we examine both the composite and each intervention individually. For example, Luth et al used literature review and expert consensus to define 5 inpatient intensive interventions: cardiopulmonary resuscitation, mechanical ventilation, intubation, feeding-tube initiation, and new dialysis.^10^ Her framework, however, does not account for the unique trajectory of postoperative recovery, where interventions may be unplanned yet potentially reversible. Accordingly, our study addressed 3 questions: (1) how often do unplanned postoperative intensive interventions occur for older adults undergoing common inpatient surgical procedures?, (2) which patients are most at risk for intensive interventions?, and (3) what are the downstream consequences once an intensive intervention has been initiated?

## METHODS

This study was approved by the Mass General Brigham institutional review board. Waiver of informed consent was obtained.

### Data Sources and Study Population

We examined Medicare fee-for-service (FFS) claims from January 1, 2016 to December 31, 2020. We utilized Medicare FFS inpatient, outpatient, home health agency, durable medical equipment, SNF, hospice, and carrier claims for the years 2016 to 2018 and MedPAR files, which were created by aggregating information for a single stay from individual inpatient and SNF claims for the years 2019 to 2020, in order to select the cohort, identify covariates, and to assess longer-term outcomes.

We identified the 10 most frequent inpatient surgeries occurring between January 1, 2017 and December 31, 2019. Diagnostic and therapeutic procedures were included, including endovascular procedures (eg, coronary stenting); however, low-risk endoscopic procedures (eg, colonoscopy) were excluded. Medicare beneficiaries 66 years or older undergoing 1 of these 10 procedures on or after January 1, 2017 to December 31, 2019 were used to create our initial cohort.

The study cohort was required to have continuous Medicare FFS enrollment during the baseline look-back period (1 year preceding the index admission date) and the follow-up period (1 year after the index date or patient death date, whichever came first). Beneficiaries were allowed to have a 1-month gap each in the look-back and follow-up periods. Those with missing information on metropolitan residence or death dates before the index date or admission type were removed.

### Primary Outcome: Intensive Interventions

Our primary outcome was the occurrence of unplanned, intensive, potentially life-sustaining interventions occurring postoperatively and during the index surgery admission. We initially generated a list of 16 intensive interventions based on previous literature and suggestions from the entire study team.^10–15^ *International Classification of Diseases,* 10th Revision (ICD-10), Procedure Coding System codes were selected based on previous studies but refined to ensure that the identified interventions were not part of routine surgical treatment and represented truly burdensome care. For example, we did not include a postsurgical ICU admission, as this is not a rare event after surgery; we clarified that transgastric and jejunal, not transnasal, feeding tubes were included; and we only considered prolonged intubation. These restrictions are likely to limit the frequency of certain procedures that may be more common in other studies (see Table 1 for codes).

**TABLE 1.** Intensive Interventions Definitions

Intervention	Operationalization	Exclusions	ICD-10-PCS Codes
Cardiopulmonary resuscitation	Any billed code on or after the date of surgery during the index admission		5A12012, 5A19054, 5A2204Z
Tracheostomy	Any billed code ≥1 d after the date of surgery during the index admission	Patients with any previous claims (1-yr lookback) with ICD-10-Dx codes Z93.0 or Z43.0, either during the index admission before the date of surgery or before the index admission, should be excluded.	0B110F4, 0B110Z4, 0B113F4, 0B113Z4, 0B114F4, 0B114Z4
ECMO	Any billed code ≥1 d after the date of surgery during the index admission		5A15223, 5A1522F, 5A1522G, 5A1522H
Prolonged renal replacement therapy (RRT)	At least 1 code billed ≥1 d after the date of surgery during the index admission, with the last instance occurring 3 d before the discharge date	Patients with any previous claims (1-yr lookback) for renal replacement therapy or ICD-10-Dx code Z99.2, either during the index admission before the date of surgery or before the index admission, should be excluded. Patients with last claim occurring >3 d from discharge date should not count as positive cases.	3E1M39Z, 5A1D70Z, 5A1D80Z, 5A1D90Z
Prolonged intubation	Any code for intubation occurring on or after the date of surgery during the index hospitalization + mechanical ventilation ≥96 h billed on or after the date of surgery during the index hospitalization	Intubation and mechanical ventilation codes do not need to have been billed on the same date. Claims for shorter periods of mechanical ventilation (5A1935Z, <24 h or 5A1945Z, 24–96 h) should not be counted as positive cases.	Intubation = 0BH17EZ or 0BH18EZ Mechanical ventilation ≥96 h = 5A1955Z
Feeding tube	Any billed code ≥1 d and during index admission after the date of surgery	Patients with any previous claims (1 yr lookback period) for feeding tube or with ICD-10-Dx codes Z93.1 or Z43.1, either during the index admission before the date of surgery or before the index admission, should be excluded.	0D16074, 0D160J4, 0D160K4, 0D160Z4, 0D163J4, 0D16474, 0D164J4, 0D164K4, 0D164Z4, 0D16874, 0D168J4, 0D168K4, 0D168Z4, 0DH60UZ, 0DH63UZ, 0DH64UZ, 0DH67UZ, 0DH68UZ, 3E0G36Z, 0DH80UZ, 0DH83UZ, 0DH84UZ, 0DH87UZ, 0DH88UZ, 0DH90UZ, 0DH93UZ, 0DH94UZ, 0DH97UZ, 0DH98UZ, 0DHA0UZ, 0DHA3UZ, 0DHA4UZ, 0DHA7UZ, 0DHA8UZ, 0DHB0UZ, 0DHB3UZ, 0DHB4UZ, 0DHB7UZ, 0DHB8UZ

We then employed a consensus-driven process whereby 8 physician authors (S.K.S., C.J.C., J.H., S.L.M., E.F., D.H.K., K.J.L., and Z.C.) formed a panel to rate the burdensomeness of the interventions, to discuss and refine the specifications, and then to re-rate a second time. The panel included physicians from vascular surgery, surgical oncology, trauma and acute care surgery, general surgery, internal medicine, geriatric medicine, pharmacoepidemiology, and health policy, providing diverse clinical perspectives on the burdensomeness of candidate interventions. Raters were asked to provide a score of 1 to 9, where 1 = low burden and 9 = high burden, and to take into consideration all of the following: the invasiveness or intensiveness of the procedure, pain and discomfort to the patient, likelihood of success, impact on longer-term outcomes, and cost to the health care system or patient. Comments were encouraged. Consensus was achieved if the median score was in the upper tercile (7–9) with no more than 3 scores in the other extreme (1–3). After the second round, of the original 16 intensive intervention candidates, a list of 6 intensive interventions was finalized, including CPR, prolonged endotracheal intubation (≥96 hours after surgery), gastrostomy/jejunostomy tube placement, tracheostomy, new dialysis, and extracorporeal membrane oxygenation (ECMO) (Table 1).

### Secondary Outcomes

Downstream outcomes of interest included mortality as an inpatient, at 90 days and 1 year; major inpatient complications; 90-day readmission; the occurrence of additional intensive interventions during follow-up; index admission length of stay; discharge home; discharge to a higher level of care; nursing home admission at 90 days; and time-at-home ratio. Major complications were defined using a list that we have previously used and that includes complications such as myocardial infarction and pneumonia.^16–18^ Several outcomes were calculated only for eligible patients. Discharge home was calculated only for community-dwelling patients, who were defined as having no MDS assessment in the 180 days before index surgery.^19^ Discharge to a higher level of care was calculated for all patients except for those coming from a long-term acute care (LTAC) facility and was defined using the following hierarchy: home < subacute nursing facility < LTAC. To determine nursing home admission, we used the MDS assessment file and excluded SNF stays using SNF claims from it.^20^ We calculated time-at-home ratio only for community-dwelling patients and calculated this as the number of days spent at home after the index surgery divided by the total follow-up days after the index surgery (maximum = 365 days). The number of days spent at home was calculated by subtracting the number of days spent in the hospital, skilled nursing facility, hospice, and LTAC facilities from total follow-up days.

### Covariates

We used the Medicare Beneficiary Summary File to collect demographic information, including vital status, sex, and race. We treated age as a continuous variable and determined race and ethnicity using the Research Triangle Institute race code embedded within this file.^21^ To define comorbid conditions, we used data from the outpatient, carrier, hospice, skilled nursing facility, home health agency, inpatient, and MedPAR files in the 1-year look-back period and summarized these using the in-hospital mortality modification of the Elixhauser comorbidity index. Case status was defined using the variables for admission source and admission type. We classified cases as urgent/emergent if the admission source was listed as the emergency room or if the admission type was listed as “emergency” or “urgent.” Rather than including indicator variables for each procedure, procedure risk was estimated as a continuous variable based on the crude in-hospital national mortality rate for each of the 10 top surgeries.

A 1-year look-back period was used to identify Alzheimer’s disease and related dementias (ADRD) and frail patients and to exclude patients who had undergone an intensive intervention before the current hospitalization. ADRD was defined using a previously validated list of ICD-10 clinical modification codes with a positive predictive value of 77.1%.^22^ Patients were dichotomized into frail versus nonfrail patients using a claims-based frailty index and a cutoff of ≥0.25.^23^ The claims-based frailty index is calculated using 93 variables derived from ICD diagnosis, current procedural terminology, fourth edition, and healthcare common procedure coding system codes.^23^ It has been validated against clinical frailty measures and outcomes including mortality, mobility impairment, and disability.^23,24^

### Statistical Analysis

We described continuous data using means, medians, interquartile range, and standard errors, and categorical data using frequencies and percentages. Unadjusted differences in outcomes were tested using Kruskal–Wallis, chi-squared, or Fisher exact test. To evaluate patients’ risk of receiving intensive interventions, we conducted a generalized estimating equation (GEE) logistic regression model, adjusting for sex, race, age, ADRD status, Elixhauser in-hospital mortality index, dual Medicare/Medicaid eligibility, frailty, metropolitan versus nonmetropolitan patient residency, procedures, and admission status (elective vs urgent/emergent) in the model. We used inverse-propensity weighting (IPW) analysis to assess the association between secondary outcomes and intensive interventions and adjusted for potential confounders. We first calculated IPW weights using a logistic regression model predicting the likelihood of the occurrence of intensive interventions. Covariates in the model for IPW weights calculation included sex, race, age, ADRD status, Elixhauser in-hospital mortality index, dual Medicare/Medicaid eligibility, frailty, metropolitan versus nonmetropolitan patient residency, procedure risk, and admission status (elective vs urgent/emergent). Next, we used IPW-weighted GEE linear regression models to assess continuous outcomes, including the time-at-home ratio and hospital length of stay. We then applied IPW-weighted GEE logistic regression models for dichotomous outcomes, including mortality, discharge home, inpatient complications, and discharge to a higher level of care. We also used multivariable GEE models to assess the association between intervention type and the outcomes by classifying beneficiaries as those having experienced no intensive interventions, a single specific intensive intervention, or multiple intensive interventions, adjusting for the same covariates included in the IPW weight calculation. Hospital clustering was accounted for in all GEE models. A 2-sided *P* value <0.05 was considered statistically significant, and a standardized mean difference ≤0.25 was considered an acceptable balance between groups in the IPW-weighted data. All analyses were performed using SAS 9.4 (SAS, Cary, NC). R 4.4.1 (R Foundation for Statistical Computing, Vienna, Austria) was used to create forest plots (https://www.r-project.org/).

## RESULTS

**FIGURE 1.**
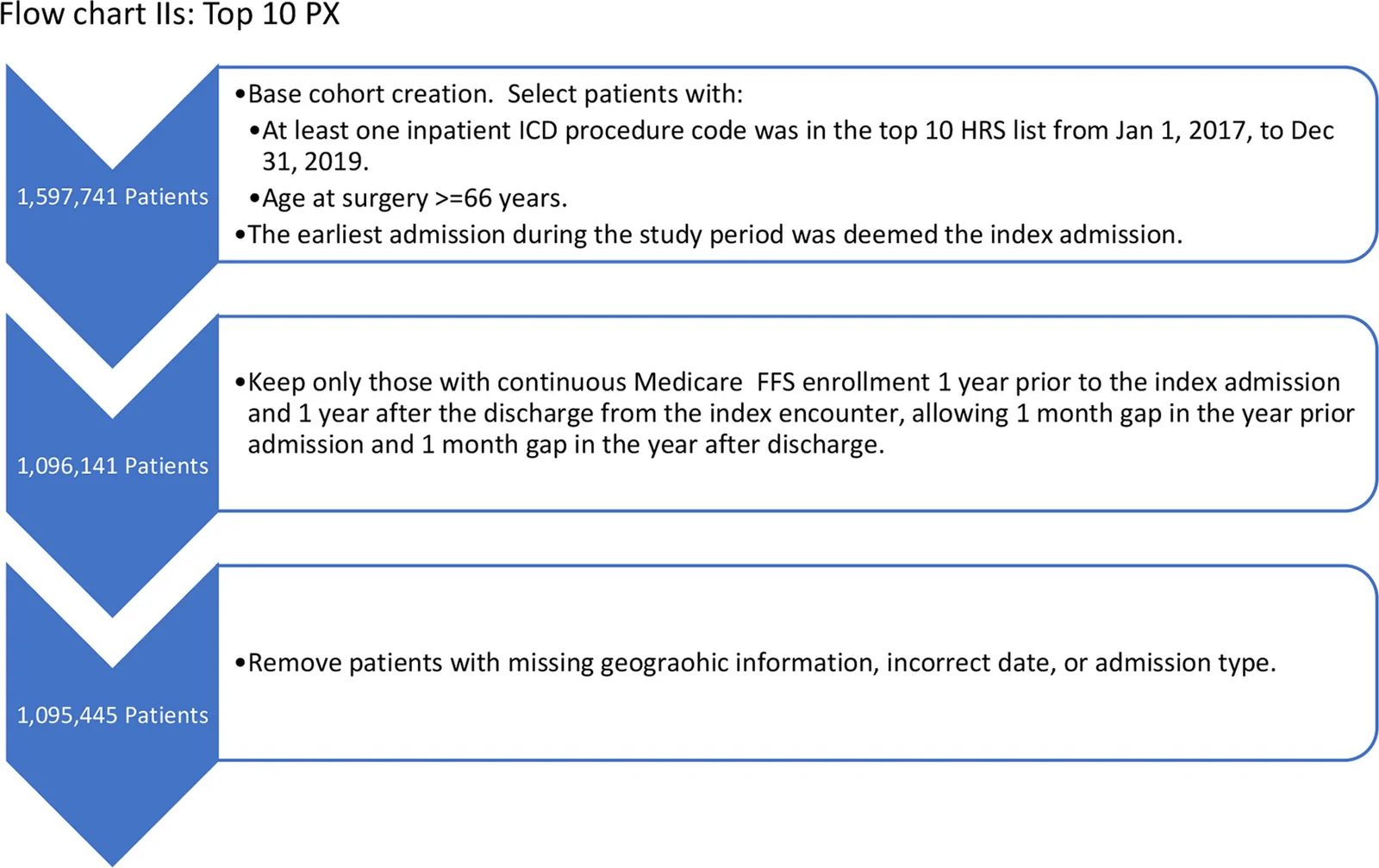
Flow diagram for cohort creation.

We identified 1,095,445 patients who underwent 1 of the 10 most common inpatient procedures (Fig. 1). The median age was 74.6 years and included 514,927 males (47.0%) (Table 2). Overall, 956,959 (87.4%) were non-Hispanic White whereas 50,143 (4.6%) and 43,378 (4.0%) were non-Hispanic Black and Hispanic, respectively. Our cohort included 825,340 (96.7%) community-dwelling patients, and 48,366 (4.4%) who had ADRD. The 3 most common significant comorbid conditions were complicated hypertension (32.6%), uncomplicated diabetes (29.0%), and chronic pulmonary disease (24.7%). Moderate/severe frailty was present in 130,494 (11.9%).

**TABLE 2.** Patient and Procedural Characteristics

		Total Population, (N = 1,095,445)		Intensive Interventions				*P*
				No		Yes		
		N or Median	% or (IQR)	N or Median	% or (IQR)	N or Median	% or (IQR)	
Age		74.6	(70.3–79.8)	74.5	(70.3–79.8)	75.9	(71.2–81.3)	<0.001
Sex	Female	580,518	53.0	573,216	98.7	7,302	1.3	<0.001
	Male	514,927	47.0	501,560	97.4	13,367	2.6	
Race	Non-Hispanic White	956,959	87.4	939,205	98.1	17,754	1.9	<0.001
	Black	50,143	4.6	49,014	97.7	1,129	2.3	
	Hispanic	43,378	4.0	42,453	97.9	925	2.1	
	Asian/native American	22,251	2.0	21,771	97.8	480	2.2	
	Unknown	15,718	1.4	15,483	98.5	235	1.5	
	Other	6996	0.6	6850	97.9	146	2.1	
Community dwelling*	Community dwelling	825,340	96.7	810,120	98.2	15,220	1.8	<0.001
Patient geography	Metropolitan	821,757	75.0	806,498	98.1	15,259	1.9	<0.001
Frail†		130,494	11.9	124,797	95.6	5697	4.4	<0.001
Elixhauser in-hospital mortality index		0	(−6 to 7)	0	(−6 to 7)	13	(2–22)	<0.001
Comorbidities								
Complicated hypertension		357,424	32.6	343,897	96.2	13,527	3.8	<0.001
Chronic pulmonary disease		270,521	24.7	263,178	97.3	7343	2.7	<0.001
Diabetes with chronic complications		237,834	21.7	229,765	96.6	8,069	3.4	<0.001
Heart failure		214,052	19.5	201,956	94.3	12,096	5.7	<0.001
ADRD		48,366	4.4	46,968	97.1	1398	2.9	<0.001
Severe renal failure		38,136	3.5	35,741	93.7	2395	6.3	<0.001
Dual eligibility		108,972	9.9	106,021	97.3	2951	2.7	<0.001
Admission urgency	Urgent/emergent	282,114	25.8	272,491	96.6	9623	3.4	<.001
Procedure risk		0.7	(0.0–1.2)	0.1	(0.0–1.2)	1.9	(1.7–1.9)	<.001

* Analysis restricted to patients who underwent surgery between January 1, 2017 and December 31, 2018.

† The CFI characteristic of dementia was not included to avoid double counting dementia.

IQR indicates interquartile range.

The procedures used to define our cohort are listed in Table 3. Of these, the 3 most common were knee arthroplasty (43.7%), coronary stenting (14.8%), and laparoscopic cholecystectomy (10.6%). Of all surgeries, 25.8% were urgent/emergent.

**TABLE 3.** Index Procedures

Total population, (N = 1,095,445)	Intensive Interventions						
	No		Yes				
	N or Median	% or (IQR)	N or Median	% or (IQR)	N or Median	% or (IQR)	*P*
Bypass of 1 coronary artery using the left internal mammary artery, open approach	69,327	6.3	64,039	92.4	5288	7.6	<0.001
Bypass of 2 coronary arteries from the aorta using autologous vein grafts, open approach	7925	0.7	7117	89.8	808	10.2	<0.001
Percutaneous dilation of 1 coronary artery with a drug eluting stent	161,103	14.7	154,448	95.9	6655	4.1	<0.001
Transcatheter (percutaneous) aortic valve replacement with a bioprosthetic valve	74,681	6.8	72,556	97.2	2125	2.8	<0.001
Laparoscopic cholecystectomy	115,708	10.6	114,415	98.9	1293	1.1	<0.001
Right shoulder joint replacement with a reverse ball and socket prosthesis, open approach	53,476	4.9	53,403	99.9	73	0.1	<0.001
Partial excision of a lumbar intervertebral disc, open approach	50,874	4.6	50,698	99.7	176	0.3	<0.001
Right hip total replacement with a ceramic on polyethylene, uncemented prosthesis, open approach	49,438	4.5	49,385	99.9	53	0.1	<0.001
Right knee total replacement with a cemented synthetic prosthesis, open approach	238,958	21.8	238,662	99.9	296	0.1	<0.001
Left knee total replacement with a cemented synthetic prosthesis, open approach	219,794	20.1	219,534	99.9	260	0.1	<0.001
Multiple procedures	54,161	4.9	50,519	93.3	3642	6.7	<0.001

IQR = interquartile range.

### Frequency of Intensive Interventions and Factors Associated with Their Occurrence

Unplanned intensive interventions occurred in 20,669 (1.9%) patients, including CPR (67.6%), prolonged intubation (22.3%), feeding tube placement (18.3%), tracheostomy (11.0%), dialysis (4.1%), and ECMO (1.9%). Among patients who experienced at least 1 intensive intervention, 3,606 (17.4%) had multiple intensive interventions.

**FIGURE 2.**
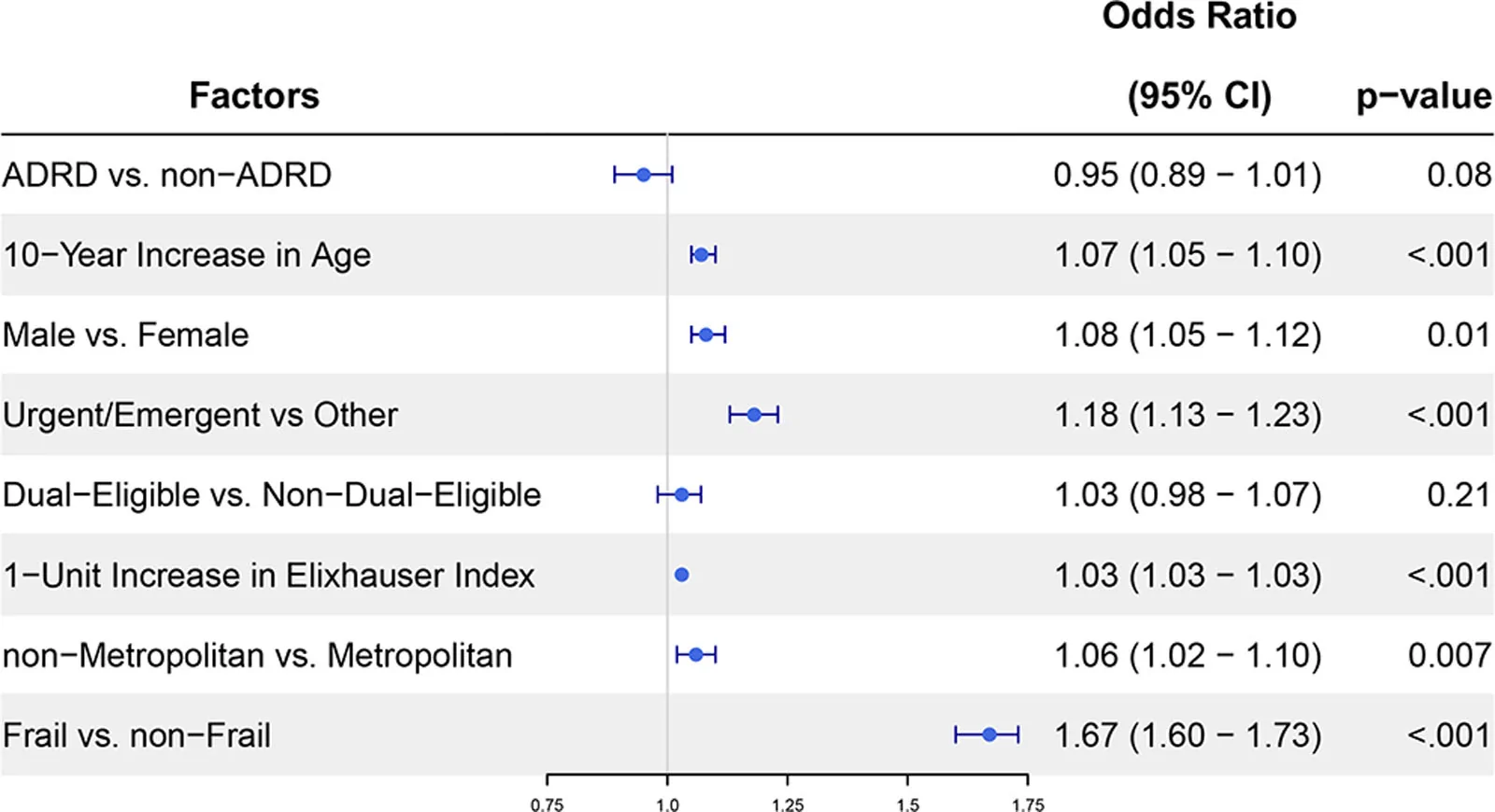
Forest plot of factors associated with the occurrence of intensive interventions after adjustment.

On adjusted analysis, male sex [adjusted odds ratio (aOR) 1.08, 95% confidence interval (CI) 1.05–1.12], increasing age (10-year increase in age (aOR 1.07, 95% CI 1.05–1.10), a higher Elixhauser inpatient mortality index (aOR 1.03, 95% CI 1.03–1.03), moderate/severe frailty (aOR 1.67, 95% CI 1.60–1.73), nonmetropolitan residency (aOR 1.06 95% CI 1.02–1.010), and urgent/emergent surgery (aOR 1.18, 95% CI 1.13–1.23) were all associated with increased odds of intensive interventions (Fig. 2).

### Outcomes After the Occurrence of Intensive Interventions

All adverse outcomes were significantly more common among patients experiencing intensive interventions compared with other surgical patients (Table 4). Notable outcomes included inpatient (24.6% vs 0.5%, *P* < 0.001) and 1-year mortality (44.2% vs 5.2%, *P* < 0.001), and inpatient major complications (67.6% vs 10.6%, *P* < 0.001). Outcomes were uniformly worse among patients experiencing intensive interventions: discharge to home (33.7% vs 78.4%, *P* < 0.001), discharge to a higher level of care (54.7% vs 21.6%, *P* < 0.001), 90-day readmissions (37.9% vs 14.9%, *P* < 0.001), 90-day nursing home admission (8.9% vs 1.8%, *P* < 0.001), time-at-home ratio (52.8 vs 93.6, *P* < 0.001), and the occurrence of additional intensive interventions (8.7% vs 1.6%, *P* < 0.001). All of these findings remained significant after adjustment using propensity score-weighted analysis (Table 4): inpatient (aOR 51.4, 95% CI 47.1–56.1) and 1-year mortality (aOR 11.6, 95% CI 10.8–12.4), inpatient major complications (aOR 15.1, 95% CI 14.0–16.2), 90-day readmissions (aOR 2.8, 95% CI 2.6–3.1), discharge to home (aOR 0.1, 95% CI 0.1–0.2) and to a higher level of care (aOR 4.8, 95% CI 4.4–5.2), 90-day nursing home admission (aOR 5.0, 95% CI 4.2–5.9), time-at-home ratio (mean difference −36.7, 95% CI −38.2 to −35.2), and the occurrence of additional intensive interventions (aOR 5.1, 95% CI 4.4–5.9).

**TABLE 4.** Frequencies and Adjusted Outcomes After Intensive Interventions

	All (N = 1,095,445)		Intensive Intervention				aOR (95% CI)	*P*
			No (N = 1,074,776)		Yes (N = 20,669)			
	N	%	N	%	N	%		
Inpatient mortality	10,919	1.0	5841	0.5	5078	24.6	51.4 (47.1–56.1)	<0.001
90-d mortality	31,179	2.8	23,524	2.2	7655	37.0	21.7 (20.2–23.4)	<0.001
1-yr mortality	65,040	5.9	55,913	5.2	9127	44.2	11.6 (10.8–12.4)	<0.001
Inpatient complication	127,631	11.7	113,665	10.6	13,966	67.6	15.1 (14.0–16.2)	<0.001
Discharge to higher level of care* (N = 1,018,886)	224,593	22.0	217,473	21.6	7120	54.7	4.8 (4.4–5.2)	<0.001
Discharge to home† (N = 825,340)	640,535	77.6	635,410	78.4	5125	33.7	0.1 (0.1– 0.2)	<0.001
30-d readmission‡ (N = 1,078,384)	94,646	8.8	91,369	8.6	3277	23.3	2.7 (2.5–3.0)	<0.001
90-d readmission‡ (N = 1,076,151)	163,401	15.2	158,170	14.9	5231	37.9	2.8 (2.6–3.1)	<0.001
Additional IIs‡ (N = 1,038,039)	17,189	1.7	16,144	1.6	1045	8.7	5.1 (4.4–5.9)	<0.001
90-d nursing home admission§ (N = 736,675)	14,141	1.9	13,353	1.8	788	8.9	5.0 (4.2–5.9)	<0.001
	Mean	SE	Mean	SE	Mean	SE	Mean Difference (95% CI)	*P*
Hospital length of stay	3.11	0.00	2.9	0.00	11.7	0.08	6.9 (6.7–11.0)	<0.001
Time-at-home ratio‖ (N = 825,335)	92.9	0.02	93.6	0.02	52.8	0.35	−36.7 (−38.2 to −35.2)	<0.001

* Patients who were admitted from the highest level of care (viz. LTAC) were removed from analysis.

† Analysis restricted to community-dwelling patients who underwent surgery between January 1, 2017 and December 31, 2018.

‡ Patients who died within the follow-up time frame and without an event of interest were removed from analysis.

§ Analysis restricted to patients discharged before December 1, 2018, October 1, 2018, and July 1, 2018 for the analysis of 30-, 90-, and 180-day NH admissions, respectively. Additionally, analyses were restricted to patients discharged alive from index admission, and patients who died within the follow-up time frame and without an event of interest were removed from analysis.

‖ Analysis restricted to community-dwelling patients who underwent surgery between January 1, 2017 and December 31, 2018. Patients with negative or over 100% time at home due to incorrect date information were removed from the analysis.

Even after restricting our analyses to patients who were discharged alive (N = 1,084,526), we found higher mortality at 90 days and 1 year: 16.6% versus 1.7% (*P* < 0.001) and 26.0% versus 4.7% (*P* < 0.001). After propensity score-weighted analysis, patients experiencing an intensive intervention continued to have higher likelihoods of mortality at both time points (90 days, aOR 9.8, 95% CI 8.9–10.7; 1 year, aOR 5.8, 95% CI 5.3–6.3).

### Outcomes by Type and Number of Intensive Interventions

We sought to further understand the association of outcomes with specific intensive interventions rather than intensive interventions as a group. When stratified by type and number of unplanned intensive interventions, outcomes varied markedly (Table 5). For example, nursing home admissions at 90 days ranged from 3.9% to 23.9% for a single intensive intervention. ECMO was associated with the highest mortality, followed by prolonged intubation (eg, ECMO inpatient mortality aOR 104.0, 95% CI 69.8–155.0; prolonged intubation aOR 38.8, 95% CI 34.2–44.1) (Supplemental Table 1, https://links.lww.com/AOSO/A637). Outcomes were especially poor among the 17.4% of patients who experienced multiple intensive interventions. For example, inpatient and 1-year mortality were 27.6% and 67.7%, and 90-day readmissions and nursing home admissions were 59.1% and 21.4%.

**TABLE 5.** Unadjusted Outcomes After the Occurrence of Individual Intensive Interventions

	All (N = 1,095,445; 100%)		No IIs (N = 1,074,776; 98.11%)		Multiple IIs (N = 3606; 0.33%)		ECMO (N = 137; 0.01%)		Feeding Tube (N = 1710; 0.16%)		Prolonged Intubation (N = 2071; 0.19%)		Resuscitation (N = 12,289; 1.12%)		Prolonged Renal Replacement Therapy (N = 562; 0.05%)		Tracheostomy (N = 294; 0.03%)	
	N	%	N	%	N	%	N	%	N	%	N	%	N	%	N	%	N	%
Inpatient mortality	10,919	1	5841	0.5	996	27.6	78	56.9	137	8	753	36.4	2800	22.8	220	39.1	94	32
90-d mortality	31,179	2.8	23,524	2.2	,965	54.5	115	83.9	505	29.5	1095	52.9	3525	28.7	299	53.2	151	51.4
1-yr mortality	65,040	5.9	55,913	5.2	2442	67.7	116	84.7	726	42.5	1212	58.5	4100	33.4	341	60.7	190	64.6
Inpatient complication	127,631	11.7	113,665	10.6	3309	91.8	115	83.9	1,289	75.4	1813	87.5	6791	55.3	370	65.8	279	94.9
90-d complication* (n = 1,087,902)	208,184	19.1	192,804	18	3486	99.2	*	*	1,478	89.4	1886	96.9	7675	66.8	441	83.1	*	*
Discharge to higher level of care† (n = 1,018,886)	224,593	22	217,473	21.6	1977	94.6	*	*	1,044	82.8	943	85.6	2788	34.6	175	61.4	*	*
Discharge to home‡ (n = 825,340)	640,535	77.6	635,410	78.4	87	3.3	*	*	153	12.8	130	8.8	4615	51	134	25.7	*	*
30-d readmission* (n = 1,078,384)	94,646	8.8	91,369	8.6	620	30.4	12	46.2	458	32.8	288	27.2	1756	19.3	94	31.8	49	29.3
90-d readmission* (n = 1,076, 151)	163,401	15.2	158,170	14.9	1109	59.1	*	*	700	51.5	465	44.5	2729	30.1	135	46.1	*	*
Additional IIs* (n = 1,038,039)	17,189	1.7	16,144	1.6	234	17.8	*	*	103	9.9	75	8.3	582	6.9	28	12	*	*
30-d nursing home admission§ (n = 808,866)	12,136	1.5	11,543	1.4	112	7.8	*	*	171	17	69	8.8	209	3.1	27	10.1	*	*
90-d nursing home admission§ (n = 736,675)	14,141	1.9	13,353	1.8	231	21.4	*	*	198	23.9	83	12.2	231	3.9	26	11.6	*	*
180-d nursing home admission§ (n = 637,875)	14,785	2.3	14,031	2.2	245	28.5	*	*	182	27.1	72	12.4	218	4.3	17	11.6	*	*
	Mean	SE	Mean	SE	Mean	SE	Mean	SE	Mean	SE	Mean	SE	Mean	SE	Mean	SE	Mean	SE
Hospital length of stay	3.11	0	2.94	0	22.67	0.27	9.88	1.28	17.14	0.27	16.48	0.21	6.74	0.05	8.07	0.29	29.57	1.42
Time-at-home ratio†	92.88	0.02	93.64	0.02	24.2	0.66	13.85	3.03	47.89	1.1	37.73	1.06	66.19	0.43	39.12	1.84	24.45	2.32

* Patients who died within the follow-up time frame and without an event of interest were removed from analysis.

† Patients who were admitted from the highest level of care (viz. LTAC) were removed from analysis.

‡ Analysis restricted to community-dwelling patients who underwent surgery between January 1, 2017 and December 31, 2018.

§ Analysis restricted to patients discharged before December 1, 2018, October 1, 2018, and July 1, 2018 for the analysis of 30-, 90-, and 180-day NH admissions, respectively. Additionally, analyses were restricted to patients discharged alive from index admission, and patients who died within the follow-up time frame and without an event of interest were removed from analysis.

## DISCUSSION

Unplanned intensive interventions have long been used as outcomes for studies of treatment at the end of life, but not as intermediate outcomes for surgery. We distilled the universe of postoperative outcomes into 6 unplanned, potentially life-sustaining but burdensome intensive interventions and found that, among >1 million Medicare beneficiaries aged ≥66 undergoing the 10 most common inpatient operations, intensive interventions were infrequent (1.9%), but strongly associated with adverse outcomes. Rather than independent causal drivers of poor outcomes, intensive interventions are best understood as sentinel markers of severe physiologic deterioration. Their occurrence was associated with dramatic increases in inpatient mortality (from 0.5% to 24.6%) and 1-year mortality (from 5.2% to 44.2%). Major complications, 90-day readmissions, and subsequent intensive interventions all similarly increased several-fold, and much higher odds of poor outcomes were present even among those who survived the hospitalization. More than 1 in 3 survivors of postoperative intensive interventions required new institutional care within 3 months of surgery. Male sex, frailty, greater comorbidity, advancing age, and urgent/emergent surgery were independently associated with increased intensive intervention risk.

Our data suggest that the prognostic meaning of intensive interventions is impacted by both the number and types of intensive interventions. Although individual interventions such as CPR or prolonged ventilation were each associated with increased mortality and institutional dependence, the combination of multiple intensive interventions was associated with especially adverse outcomes including 27.6% inpatient mortality, fewer than 5% of patients discharged home, and 21.4% nursing home admissions at 90 days. Importantly, although the 6 individual intensive interventions vary considerably in mechanism, invasiveness, and reversibility—ranging from feeding tube placement to ECMO—they share a defining attribute: each represents an unplanned, potentially life-sustaining intervention that was not part of the expected surgical course and that imposes substantial burden on the patient, which is consistent with the measures found in the literature on intensity of treatment at the end of life.^11^ Nevertheless, in the context of surgery, the composite is best understood as a population-level epidemiologic construct for identifying patients whose postoperative trajectory has been fundamentally altered, rather than as an assertion of clinical equivalence among the constituent interventions. For patient-level counseling, the individual intervention-specific outcomes presented in Table 5 and the Supplemental Table 1, https://links.lww.com/AOSO/A637 should be used, as they capture the wide range of prognoses (eg, inpatient mortality of 8.0% for feeding tube alone vs 56.9% for ECMO).

Our intensive intervention list overlaps with, but deliberately narrows, the end-of-life framework proposed by Luth et al (CPR, mechanical ventilation, intubation, feeding-tube initiation, and new dialysis), adapting it to the distinctly postoperative setting, where interventions may be unplanned yet for reversible conditions.^10^ The 1.1% incidence of CPR we observed is higher than the 0.36% postoperative CPR rate reported in a large 2008 to 2012 ACS-NSQIP study of mixed-age adults—likely reflecting our focus on older, more frail Medicare patients.^25^ Our outcomes after a single intensive intervention mirrored those described in contemporary work on perioperative CPR, where 30-day mortality exceeded 60% and frailty magnified risk.^26^

Our data contribute in 2 ways. First, the identification of patient-level risk factors—particularly frailty (aOR 1.67), comorbidity burden, and urgent/emergent surgical status—can inform preoperative shared decision-making by helping clinicians and patients anticipate the possibility of these events. Second, and perhaps more importantly, the occurrence of a postoperative intensive intervention should be understood as a sentinel event. The dramatic downstream consequences we observed—including 44% 1-year mortality, a 37-point reduction in time-at-home ratio, and fivefold increased odds of nursing home admission—argue that an intensive intervention should trigger early, structured reassessment of goals of care with patients and caregivers. There are several important limitations to our approach. First, our consensus-based approach to identifying burdensome intensive interventions did not involve patients’ lived experience or caregivers—their perspective is crucial and may have resulted in a different list of interventions. Second, we lacked access to code status and its changes throughout the clinical course and discussions surrounding care, which influence outcomes. For example, deaths from decisions to withdraw life-sustaining treatment in a patient on ECMO would be indistinguishable from deaths in patients who are full-code status. Third, we were unable to assess outcomes important to patients and caregivers such as caregiver burden, cognitive function, mobility, pain, and other quality of life indicators. Fourth, the occurrence of an unplanned intensive intervention is likely to be associated with other complications—for example, pneumonia associated with respiratory failure leading to ECMO. Our data, however, do not include timestamps that would allow us to distinguish between complications leading to intensive interventions and complications occurring with or after intensive interventions. Accordingly, causality between intensive interventions and observed outcomes cannot be established; intensive interventions should be interpreted as markers of catastrophic physiologic deterioration rather than independent drivers of mortality. Last, our data include only FFS Medicare beneficiaries aged 66 years and older—extrapolation to other populations should be done with caution.

Our study is advantaged in several important respects. Our data are national in scope and avoid the disadvantages of data drawn from a limited number of centers or surgeons. The scope of our procedures is broad and focused on the most common surgeries rather than single procedures or rarer high-risk procedures, with a corresponding increase in the number of patients to whom our data directly apply. Our overall construct is patient-centered with a focus on intensive interventions as those interventions that impose an undue burden on patients. Although cardiac procedures (coronary artery bypass grafting, percutaneous coronary intervention, and transcatheter aortic valve replacement) contributed a disproportionate share of intensive interventions given their higher event rates, our multivariable models adjust for procedure risk, and the associations between patient-level factors and intensive interventions remain robust after this adjustment. Examining whether frailty modifies intensive intervention risk differentially across surgical specialties is an important direction for future investigation.

## CONCLUSIONS

Although postoperative unplanned intensive interventions are rare, they are associated with a marked worsening in survival, likelihood of institutionalization, and other undesirable outcomes for older adults. Frailty, higher burdens of comorbidities, older age, and urgent/emergent surgery status were associated with increased risks of intensive intervention. Multiple intensive interventions generally amplified the magnitude of these outcomes. The occurrence of a postoperative intensive intervention should be recognized as a sentinel event associated with dramatically worsened survival, high rates of institutionalization, and reduced time at home. These data support incorporating patient-level risk factors into preoperative shared decision-making and, critically, using an intensive intervention as a trigger for structured reassessment of goals of care, palliative care involvement, and transition planning.

## SPONSOR’s ROLE

Research reported in this publication was supported by the National Heart, Lung, and Blood Institute of the National Institutes of Health under Award Number NIA R01AG067507. The content is solely the responsibility of the authors and does not necessarily represent the official views of the National Institutes of Health.

## DATA ACCESS

The data used in this study are available from the Centers for Medicare and Medicaid Services (CMS) Chronic Conditions Warehouse. Due to data use agreement restrictions, the analytic dataset cannot be shared directly. The analytic code is available from the corresponding author upon reasonable request.

## Supplementary Material


