# Supplementary material for: Incidence and Outcomes of Unplanned Intensive Interventions After Common Inpatient Surgeries: A Population-Based Cohort Study
Source: Ann Surg Open. 2026 Jul 3;7(3):e692. doi: 10.1097/AS9.0000000000000692 (PMC13596944; doi:10.1097/AS9.0000000000000692)
Supplement: Supplementary file 1 [file as9-7-e692-s001.pdf]

Supplemental Table

Supp Table 1. Adjusted outcomes after the occurrence of individual intensive interventions

Outcome	Intensive Intervention	OR (95% CI)	P-value
**Inpatient Mortality**	No Intensive Intervention	REF	
	Multiple Intensive Interventions	21.4 (19.3 - 23.8)	<.001
	ECMO	104.0 (69.8 - 155.0)	<.001
	Feeding Tube	4.6 (3.8 - 5.6)	<.001
	Prolonged Intubation	38.8 (34.2 - 44.1)	<.001
	Resuscitation	23.0 (21.5 - 24.7)	<.001
	Prolonged Renal Replacement Therapy	37.6 (29.8 - 47.5)	<.001
	Tracheostomy	28.0 (20.7 - 37.9)	<.001
**90-Day Mortality**	No Intensive Intervention	REF	
	Multiple Intensive Interventions	22.3 (20.5 - 24.2)	<.001
	ECMO	162.2 (99.7 - 263.8)	<.001
	Feeding Tube	6.1 (5.4 - 7.0)	<.001
	Prolonged Intubation	23.0 (20.5 - 25.9)	<.001
	Resuscitation	9.2 (8.7 - 9.7)	<.001
	Prolonged Renal Replacement Therapy	19.7 (15.8 - 24.6)	<.001
	Tracheostomy	20.4 (15.4 - 27.2)	<.001
**1-Year Mortality**	No Intensive Intervention	REF	
	Multiple Intensive Interventions	17.1 (15.7 - 18.6)	<.001
	ECMO	75.6 (45.8 - 124.9)	<.001
	Feeding Tube	4.5 (4.0 - 5.1)	<.001
	Prolonged Intubation	11.9 (10.6 - 13.4)	<.001
	Resuscitation	4.8 (4.6 - 5.1)	<.001
	Prolonged Renal Replacement Therapy	11.3 (9.0 - 14.1)	<.001
	Tracheostomy	15.7 (11.7 - 20.9)	<.001
**Inpatient Complication**	No Intensive Intervention	REF	
	Multiple Intensive Interventions	34.7 (30.2 - 39.8)	<.001
	ECMO	16.7 (10.2 - 27.3)	<.001
	Feeding Tube	11.6 (10.2 - 13.3)	<.001
	Prolonged Intubation	27.3 (23.4 - 31.8)	<.001
	Resuscitation	4.5 (4.3 - 4.7)	<.001
	Prolonged Renal Replacement Therapy	6.4 (5.3 - 7.8)	<.001
	Tracheostomy	61.7 (36.3 - 104.7)	<.001
**Discharge to Higher Level of Care[2]**	No Intensive Intervention	REF	
	Multiple Intensive Interventions	92.9 (75.7 - 114.1)	<.001
	ECMO	129.0 (39.9 - 417.5)	<.001

	Feeding Tube	20.2 (17.0 - 23.9)	<.001
	Prolonged Intubation	31.1 (25.9 - 37.3)	<.001
	Resuscitation	2.7 (2.5 - 2.8)	<.001
	Prolonged Renal Replacement Therapy	7.2 (5.6 - 9.4)	<.001
	Tracheostomy	114.3 (52.6 - 248.7)	<.001
**Discharge to Home[3]**	No Intensive Intervention	REF	
	Multiple Intensive Interventions	0.0 (0.0 - 0.0)	<.001
	ECMO	0.0 (0.0 - 0.0)	<.001
	Feeding Tube	0.0 (0.0 - 0.0)	<.001
	Prolonged Intubation	0.0 (0.0 - 0.0)	<.001
	Resuscitation	0.2 (0.2 - 0.2)	<.001
	Prolonged Renal Replacement Therapy	0.1 (0.1 - 0.1)	<.001
	Tracheostomy	0.0 (0.0 - 0.0)	<.001
**30-Day Readmission[1]**	No Intensive Intervention	REF	
	Multiple Intensive Interventions	1.9 (1.7 - 2.0)	<.001
	ECMO	5.0 (2.3 - 10.9)	<.001
	Feeding Tube	2.3 (2.0 - 2.6)	<.001
	Prolonged Intubation	1.8 (1.6 - 2.1)	<.001
	Resuscitation	1.4 (1.4 - 1.5)	<.001
	Prolonged Renal Replacement Therapy	2.0 (1.6 - 2.7)	<.001
	Tracheostomy	1.9 (1.3 - 2.7)	0.001
**90-Day Readmission[1]**	No Intensive Intervention	REF	
	Multiple Intensive Interventions	3.4 (3.1 - 3.8)	<.001
	ECMO	4.9 (2.1 - 11.4)	<.001
	Feeding Tube	2.7 (2.4 - 3.0)	<.001
	Prolonged Intubation	2.1 (1.8 - 2.4)	<.001
	Resuscitation	1.4 (1.3 - 1.5)	<.001
	Prolonged Renal Replacement Therapy	2.0 (1.5 - 2.5)	<.001
	Tracheostomy	2.5 (1.8 - 3.6)	<.001
**Additional Intensive Interventions[1]**	No Intensive Intervention	REF	
	Multiple Intensive Interventions	4.4 (3.8 - 5.2)	<.001
	ECMO	5.7 (1.8 - 18.4)	0.004
	Feeding Tube	2.4 (1.9 - 3.0)	<.001
	Prolonged Intubation	2.1 (1.6 - 2.7)	<.001
	Resuscitation	2.3 (2.1 - 2.6)	<.001
	Prolonged Renal Replacement Therapy	2.6 (1.7 - 4.0)	<.001
	Tracheostomy	4.0 (2.4 - 6.5)	<.001
**90-Day Nursing Home Admissions[4]**	No Intensive Intervention	REF	
	Multiple Intensive Interventions	8.9 (7.6 - 10.5)	<.001
	ECMO	5.1 (0.6 - 46.5)	0.15
	Feeding Tube	8.9 (7.3 - 10.8)	<.001
	Prolonged Intubation	4.5 (3.4 - 5.8)	<.001
	Resuscitation	1.7 (1.5 - 1.9)	<.001
	Prolonged Renal Replacement Therapy	3.6 (2.3 - 5.6)	<.001
	Tracheostomy	7.6 (4.3 - 13.7)	<.001

[1] Patients who died within the follow-up time frame and without event of interest were removed from analysis.

[2] Patients who admitted from the highest level of care (viz. LTAC) were removed from analysis.

[3] Analysis restricted to community-dwelling patients underwent surgery between 01/01/2017 to 12/31/2018.
[4] Analysis restricted to patients discharged before 12/01/2018, 10/01/2018, and 07/01/2018 for the analysis of 30-, 90-, and 180-day NH admissions, respectively. Additionally, analyses were restricted to patients discharged alive from index admission, and patients who died within the follow-up time frame and without an event of interest were removed from analysis.
